# Neurotransmission on Fire?: Metabolic Activation Heightens Effect of PBDEs

**Published:** 2008-05

**Authors:** Julia R. Barrett

Polybrominated diphenyl ethers (PBDEs) are man-made flame retardants used in a variety of consumer products, including fabrics, cushion foam, carpet pads, computers, and electronic equipment. Ubiquitous in the environment, PBDEs and their metabolites are being found in wildlife and humans, but little is known about the human health effects of these chemicals. A new study now reveals that 6-OH-BDE-47, a hydroxylated metabolite of the widely used BDE-47, induces a more potent response than the parent compound in terms of intracellular calcium concentration (denoted [Ca^2+^]*_i_*) and release of catecholamine neurotransmitters **[*EHP* 116:637–643; Dingemans et al.]**. This finding suggests that metabolic activation could increase the neurotoxic potential of PBDEs.

[Ca^2+^]*_i_* controls biological processes such as neurotransmitter release by fluctuating in response to chemical and electrical signals. In cell culture studies, high concentrations of PBDEs induced an increase in intracellular Ca^2+^. *In vitro* endocrine studies have further revealed that 6-OH-BDE-47 interacts more strongly with hormone receptor systems than its parent compound. Preliminary evidence suggests that exposure to high concentrations of PBDEs may cause neurobehavioral alterations and affect the immune system in animals. Exposure of newborn mice to high concentrations of BDE-47 alters behavior, learning and memory, and brain protein density and enzyme activity.

The authors used cultured rat pheochromocytoma (PC12) cells, which secrete catecholamines upon stimulation, to compare the effects of 6-OH-BDE47 with those of BDE-47 on intracellular Ca^2+^ balance and catecholamine release. Members of the team had earlier measured the frequency of vesicular catecholamine release in individual cells in response to 6-OH-BDE-47 exposure and found that a high concentration of BDE-47 (20 μM) could trigger catecholamine release in PC12 cells. In the current study, they found that a lower concentration (5 μM) of 6-OH-BDE-47 caused an even stronger response. Moreover, 1 μM 6-OH-BDE-47 caused an initial transient increase in [Ca^2+^]*_i_* that was temporally related to catecholamine release. At 6-OH-BDE-47 concentrations of 1 μM or higher, they observed a delayed, dose-dependent increase in [Ca^2+^]*_i_*. Additional experiments revealed that the initial increase originated from emptying of the endoplasmic reticulum, whereas the delayed increase originated primarily from mitochondria.

Based on these findings, the authors conclude that 6-OH-BDE-47 can disrupt intracellular Ca^2+^ levels and trigger neurotransmitter release at lower levels than its parent compound, BDE-47. This conclusion suggests the neurotoxic potential of PBDEs may be strongly enhanced by oxidative metabolism, which is especially relevant for children, who may be exposed to higher levels of PBDE than adults at a time when their brains are still developing. Given recent findings that hydroxylated PBDE metabolites, including 6-OH-BDE-47, bioaccumulate in the serum of children, further investigation of potential neurotoxicity is vital.

